# Alcohol Worsens Acute Lung Injury by Inhibiting Alveolar Sodium Transport through the Adenosine A1 Receptor

**DOI:** 10.1371/journal.pone.0030448

**Published:** 2012-01-17

**Authors:** Laura Dada, Angel R. Gonzalez, Daniela Urich, Saul Soberanes, Tomas S. Manghi, Sergio E. Chiarella, Navdeep S. Chandel, G. R. Scott Budinger, Gökhan M. Mutlu

**Affiliations:** Department of Medicine, Division of Pulmonary and Critical Care Medicine, Northwestern University Feinberg School of Medicine, Chicago, Illinois, United States of America; Johns Hopkins School of Medicine, United States of America

## Abstract

**Objective:**

Alcohol intake increases the risk of acute lung injury (ALI) and the acute respiratory distress syndrome (ARDS) and is associated with poor outcomes in patients who develop these syndromes. No specific therapies are currently available to treat or decrease the risk of ARDS in patients with alcoholism. We have recently shown increased levels of lung adenosine inhibit alveolar fluid clearance, an important predictor of outcome in patients with ARDS. We hypothesized that alcohol might worsen lung injury by increasing lung adenosine levels, resulting in impaired active Na^+^ transport in the lung.

**Methods:**

We treated wild-type mice with alcohol administered i.p. to achieve blood alcohol levels associated with moderate to severe intoxication and measured the rate of alveolar fluid clearance and Na,K-ATPase expression in peripheral lung tissue and assessed the effect of alcohol on survival during exposure to hyperoxia. We used primary rat alveolar type II cells to investigate the mechanisms by which alcohol regulates alveolar Na^+^ transport.

**Results:**

Exposure to alcohol reduced alveolar fluid clearance, downregulated Na,K-ATPase in the lung tissue and worsened hyperoxia-induced lung injury. Alcohol caused an increase in BAL fluid adenosine levels. A similar increase in lung adenosine levels was observed after exposure to hyperoxia. In primary rat alveolar type II cells alcohol and adenosine decreased the abundance of the Na,K-ATPase at the basolateral membrane via a mechanism that required activation of the AMPK.

**Conclusions:**

Alcohol decreases alveolar fluid clearance and impairs survival from acute lung injury. Alcohol induced increases in lung adenosine levels may be responsible for reduction in alveolar fluid clearance and associated worsening of lung injury.

## Introduction

Acute lung injury (ALI) and ARDS are life-threatening conditions that affect almost 200,000 people in the United States every year, accounting for 3.6 million hospital days and resulting in ∼75,000 deaths [1]–[3]. Patients who chronically use alcohol have a two- to four-fold higher risk for the development of ALI/ARDS and worse outcomes when they develop ARDS [4], [5]. The molecular mechanisms underlying this association are incompletely understood and no specific therapies are currently available to treat or decrease the risk of lung injury in patients with alcoholism.

Pathologically, ARDS is characterized by damage to the alveolar-capillary barrier resulting in the accumulation of edema fluid in the alveolar space. This fluid impairs gas exchange, resulting in hypoxemia and respiratory failure. Resolution of ALI/ARDS requires clearance of excess alveolar edema fluid and repair of the alveolar capillary barrier [6], [7]. A major function of the alveolar epithelium is the clearance of edema fluid via the active transport of Na^+^ across the alveolar epithelium to the blood through apically-localized Na^+^ channels (ENaC) down a gradient generated by basolateral membrane-localized Na,K-ATPase pumps. Most patients with ALI/ARDS have impaired alveolar fluid clearance (AFC) and those who cannot augment their rates of AFC after pharmacologic stimulation have worse outcomes [8]. We and others have shown that strategies designed to maintain or enhance AFC by upregulation of the Na,K-ATPase decrease the severity of ALI and improve survival in animals and humans with ALI/ARDS [9]–[16].

Both acute and chronic ingestion of alcohol causes an increase in the systemic levels of extracellular adenosine via inhibition of the nucleoside transporter, which impairs the uptake of adenosine [17]–[20]. We have previously reported that adenosine causes a dose-dependent reduction in AFC through stimulation of the of the adenosine type 1 receptor (ADORA1) [21]. In this study, we sought to determine whether an alcohol mediated increase in adenosine might impair alveolar fluid clearance and worsen acute lung injury.

## Methods

### Animals and induction of acute lung injury

The protocol for the use of mice (ASP-2009-1041 and ASP-2009-1585) was approved by the Animal Care and Use Committee at Northwestern University. We used eight to twelve week old, (20-25 g), male, C57BL/6 mice (Charles River). For induction of non-infectious or infectious ALI, we exposed mice to either hyperoxia or to intratracheal influenza A, respectively. To induce hyperoxic ALI, mice were exposed to normobaric hyperoxia (100% O_2_) in a Kirschner animal chamber for up to 10 days as we have previously described (11).

### Administration of ethanol

We administered ethanol (4g/kg, 20% v/v in sterile water i.p.) or an equivalent volume of sterile water to mice daily once daily starting 3 days after prior to measurement of alveolar fluid clearance or the induction of acute lung injury [22]. We continued ethanol or control vehicle (sterile water) administration for two additional days after the initiation of exposure to hyperoxia for a total duration of 5 days.

### Measurement of alveolar fluid clearance (AFC)

The rate of AFC was measured as we previously described [2], [12], [23]. Briefly, mice are anesthetized with diazepam (5 mg/kg, i.p.) to decrease anxiety related catecholamine release followed 10 minutes later by pentobarbital (50–75 mg/kg, i.p.). After complete sedation was achieved, a tracheostomy tube was inserted and the animals were attached to a mechanical ventilator (Harvard Apparatus MiniVent) and ventilated at a rate of 200 breaths per minute with a V_T_ of 150 µL, FiO_2_ of 1.0 and PEEP of 2 cm H_2_O. Pancuronium (0.02 mg i.p.) was administered and after the cessation of respiratory efforts, 300 µL of iso-osmotic 5% albumin (324 mOsm/L) with Evans Blue dye (0.15 mg/ml) was instilled into the tracheostomy tube. The animals were ventilated for 30 minutes after which fluid is aspirated from the tracheostomy tube. The concentration of Evans Blue labeled albumin was measured using a spectrophotometer and AFC was calculated as the percentage increase in Evans Blue concentration over the 30 minute time frame as previously described [2].

### Measurement of adenosine levels

Plasma and bronchoalveolar lavage (BAL) fluid levels of adenosine were measured in mice 4 hours after exposure to alcohol (4 g/kg, 20% v/v in sterile water i.p.), or control vehicle (sterile water) using an HPLC as we previously described [21]. Blood was collected via right atrial puncture into a syringe containing sodium citrate (final concentration 3.2%) and the adenosine deaminase inhibitor, erythro-9-(2-hydroxy-3-nonyl)adenine hydrochloride (EHNA; 2.5 µM) and the nucleotide transport inhibitor dipyridamole (250 µM) (stopping solutions) then centrifuged (1200 rpm, 5 minutes) to isolate the plasma fraction. PBS containing the stopping solutions (0.8 ml) was instilled and aspirated into the lungs three times. The resulting bronchoalveolar lavage fluid was centrifuged (1200 rpm, 5 minutes) and adenosine measured in the supernatant.

### Assessment of the generation of reactive oxygen species (ROS)

Miotchondrial ROS generation was measured using cells stably expressing an oxidant sensitive GFP probe containing a mitochondrial localization sequence (mito-Ro-GFP). This probe was originally described by Hanson and colleagues who validated its responsiveness to a variety of intracellular oxidants both *ex vivo* and in living cells [17]. The sequence for the RoGFP probe with a mitochondrial localization sequence was cloned into a lentiviral vector (Virapower Lentiviral Expression System, Invitrogen) and was expressed in packaging cells as previously described and was used to generate MLE 12 cells (catalog no CRL-2110, ATCC, Manassas, VA) stably expressing the probe [24]. Localization of the probe to the mitochondrial matrix was confirmed by confocal microscopy after staining the cells with MitoTracker (10 µM, 10 minutes in the dark) (Invitrogen) [24]. Oxidation of the mito-Ro-GFP probe was assessed using flow cytometry. After treatment, the cells were removed from the plate using trypsin and equal aliquots of the resulting suspension were transferred to tubes containing media alone or media containing 1mM Dithiothreitol (DTT) or 1 mM t-butyl hydroperoxide (TBHP). After 10 minutes, the ratio of fluorescence (emission of 535 nm) at excitations of 400 and 490 nm was be measured in 5,000 cells per condition using a DakoCytomation CyAn high speed multilaser droplet cell sorter. The oxidation state of the cells was calculated as the completely reduced ratio (DTT) less the untreated value divided by the difference in the ratio observed with DTT and TBHP.

### Isolation and culture of primary rat type II alveolar epithelial cells (AEC)

Primary type II AEC were isolated from the lungs of Sprague-Dawley rats weighing 200–225 g, as we have previously described [25]–[27]. The day of isolation and plating was designated culture day 0. All experiments were conducted on days 2 or 3. All cells were incubated in a humidified atmosphere of 5% CO_2_/95% air at 37°C.

### Biotinylation of cell surface proteins

After the corresponding treatment, cells were placed on ice, washed twice with ice-cold PBS, and surface proteins were labeled for 20 minutes using 1 mg/ml EZ-link NHS-SS-biotin (Pierce Chemical Co) following the protocol by Gottardi and coworkers [28]. After labeling, the cells were rinsed three times with PBS containing 100 mM glycine to quench unreacted biotin, and then lysed in modified RIPA buffer (mRIPA: 50 mM Tris-HCl, pH 8, 150 mM NaCl, 1% NP-40 and 1% sodium deoxycholate and protease inhibitors). Proteins (75 µg) were incubated overnight at 4°C with end-overend shaking in the presence of Streptavidin beads (Pierce Chemical Co). Beads were thoroughly washed, resuspended in 30 µl of Laemmli's sample buffer solution and analyzed by western blot with a Na,K-ATPase α_1_ subunit specific antibody (clone 464.6; 1∶10,000, Milipore).

### Collection of peripheral lung tissue and isolation of basolateral cell membrane

Basolateral plasma membrane proteins were obtained by homogenizing lung tissue collected from the peripheral 1–2 mm of each lobe as previously described [15], [29].

### Cell lysis and western blot analysis

After treatment, AEC were washed in ice-cold PBS and solubilized in lysis buffer (20 mM Tris-HCl, pH 7.4, 150 mM NaCl, 1 m M EGTA, 1 mM Na_2_EDTA, 1% Triton X-100, 2.5 sodium pyrophosphate, 1mM b-glycerolphosphate, 1 mM Na_3_VO_3_, and protease inhibitors). The lysates were cleared by centrifugation for 10 min at 14,000 x *g*. Protein concentrations were determined by Bradford assay using a commercial dye reagent (Bio-Rad) and samples containing equal amounts (50 to 75 µg) of proteins were separated by SDS-PAGE and transferred onto nitrocellulose membranes (Optitran; Schleider & Schuell) by using a semi-dry transfer apparatus (Bio-Rad). The following commercially available antibodies and dilutions were used for western blotting: rabbit anti-pAMPKá (T172) and anti-AMPKá were from Cell Signaling Technology and were used at 1∶1,000; mouse anti-HA-tag (clone 16B12, 1∶1000) was from Covance, mouse anti-Na,K-ATPase subunit á_1_ (clone 464.6; 1∶10,000) was from Upstate Biotechnology. Primary antibodies were detected by horseradish peroxidase-conjugated secondary goat anti-mouse antibodies (1∶10,000; Bio-Rad) or goat anti-rabbit antibodies (1∶2,000; Cell Signaling Technology) by using chemiluminescence detection kit (PerkinElmer Life Sciences). Quantification of protein levels was performed by densitometric scanning with ImageJ 1.29X (NIH).

### Adenoviral infection of primary rat AEC

Day 2 type II rat AEC, plated on 60-mm cell culture dishes were incubated with an adenovirus without cDNA (Ad-null; 20 or 50 pfu/cell) or with an adenovirus carrying a dominant-negative, kinase dead (K45R) variant of the AMPK-á_1_-subunit (Ad-DN–AMPK-α1; 20 pfu/cell), a generous gift of Dr Lee Witters (Dartmouth University) for 2–4 h in 500 µl DMEM [30]. After the 2- to 4-h incubation period, 1.5 ml of DMEM supplemented with 10% fetal bovine serum, 100 U/ml penicillin and 100 µg/ml streptomycin was added to the cell culture plates and experiments were performed 24 h later.

### Measurement of the concentration of adenine nucleotides in AEC

Primary rat type II AEC were incubated in 300 µl of media containing 20 µl of 1M HClO_4_ and centrifuged at maximum speed for min. HClO_4_ was removed by mixed phase extraction employing 11.75:13.25 (v:v) of tri n-octylamine and Freon 11. Concentrations of adenine nucleotides in alveolar epithelial cell lysates were analyzed with a Hitachi D-7000 HPLC equipment with a Supelcosil C18 column (Sigma) with an elution flow rate of 1 ml/min in HPLC grade methanol, H_2_O, buffer B [50 mM KH_2_PO_4_, 8 mM tetrabutylammonium hydrogen sulfate (TBAS) and 40% (v:v) acetonitrile at pH 5.8], and buffer A [50.0 mM KH_2_PO_4_, and 8 mM TBAS at pH 5.8] as previously described [31]. Injection volume was 100 µl. A linear gradient of 15 min from 10% buffer B, 90% buffer A to 45% buffer B, 55% buffer A was employed to elute nucleotides in a total analysis time of 30 min. Areas under the AMP, ADP and ATP peaks were quantified with the use of an electronic integrator and in comparison to the standard curves created for each adenosine nucleotide at 254 nm.

### Statistics

Differences between groups were explored using analysis of variance. When the analysis of variance indicated a significant difference, individual differences were explored using t tests with a Dunnett correction for multiple comparisons against control conditions. All of the analyses were performed using GraphPad Prism version 4.00 for Windows (GraphPad Software, San Diego, CA).

## Results

### Alcohol increases mortality from hyperoxia-induced acute lung injury in mice

**Figure 1:**
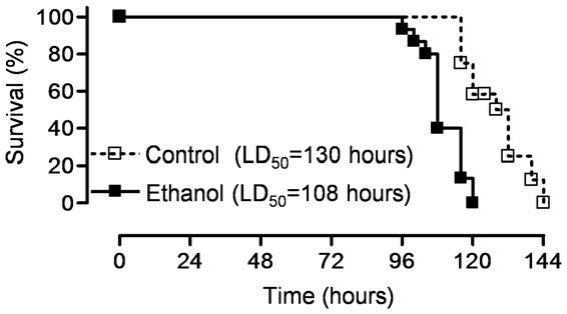
Alcohol accelerates mortality from hyperoxia-induced acute lung injury in mice. Mice were treated with ethanol (20% v/v, 4 g/kg i.p.) or control vehicle (sterile water) daily starting 3 days prior to and during the first 2 days of exposure to hyperoxia (>95% O_2_) for a total of 5 days. The time when 50% of the animals had died (LD_50_) was calculated. (N = 8/group for each treatment group).

We treated mice with ethanol (20% v/v, 4 g/kg i.p.) or an identical volume of sterile water daily starting 3 days prior to and during the first 2 days of exposure to normobaric hyperoxia, a well-described model of acute lung injury. Compared with vehicle-treated mice, alcohol-treated mice had decreased survival (LD_50_ = 130 hours and 108 hours, respectively, P = 0.0037) (Figure 1).

### Alcohol inhibits alveolar fluid clearance and active Na^+^ transport

**Figure 2:**
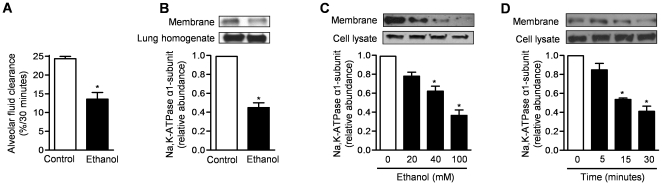
Alcohol decreases the rate of alveolar fluid clearance and downregulates Na,K-ATPase *in vivo* and *in vitro*. Mice were treated with ethanol (20%, 4 g/kg i.p.) or control vehicle (sterile w) daily for 5 days and (**A**) alveolar fluid clearance was measured and (**B**) basolateral membrane (BLM) abundance of α_1_-subunit of the Na,K-ATPase in peripheral lung tissue was evaluated via immunoblotting 4 hours after the last dose. N = 7 mice/group for alveolar fluid clearance measurements and N = 3 for evaluation of Na,K-ATPase, *p<0.05 between alcohol and control vehicle. (**C, D**) Plasma membrane abundance of the α_1_-subunit of the Na,K-ATPase in primary rat AEC was evaluated via immunoblotting (**C**) 30 minutes after treatment with different doses of ethanol and (**D**) for up to 30 minutes after treatment with high dose ethanol (100mM). N≥3 for all measures, *p<0.05 for comparison with untreated cells.

To evaluate the effect of alcohol on alveolar epithelial Na^+^ transport, we measured AFC in mice treated with either alcohol (20% v/v, 4g/kg, i.p.) or sterile water daily for up to 5 days and measured the AFC 4 hours after the last dose. Exposure to alcohol for 5 days was associated with a ∼40% reduction in alveolar fluid clearance compared to control treatment (Figure 2A). We then performed immunoblotting against α_1_-subunit of Na,K-ATPase in the peripheral lung tissue (distal 1-2 mm of each lobe). The expression of Na,K-ATPase in the basolateral membrane (BLM) relative to the whole lung epithelial cells (lung homogenate) was determined (Figure 2B).

To evaluate whether the alcohol-induced reduction in AFC is mediated via its effect on transepithelial active Na^+^ transport in the alveolar epithelium, we treated primary rat AEC with increasing doses of ethanol (0-100 mM) and measured the basolateral membrane abundance of the α_1_-subunit of Na,K-ATPase via immunoblotting (Figure 2C). We also measured plasma membrane abundance of α_1_-subunit of Na,K-ATPase at different time points after treatment with high dose ethanol (100 mM) (Figure 2D). We found that alcohol caused a dose- and time-dependent reduction in the membrane abundance of the Na,K-ATPase in AEC.

### Alcohol does not cause the generation of reactive oxygen species (ROS) in alveolar epithelial cells

**Figure 3:**
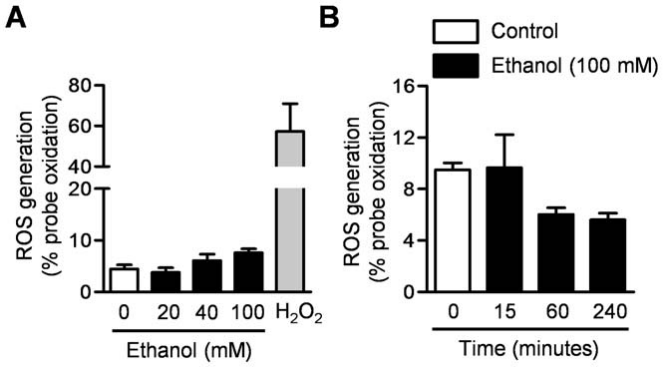
Alcohol does not increase ROS generation in alveolar epithelial cells. (**A**) MLE-12 cells that stably express a mitochondrially localized oxidant sensitive GFP probe were treated with different doses of ethanol (0-100 mM), media (negative control) or H_2_O_2_ (positive control) and ROS generation was measured 4 hours later. (**B**) The same cells were treated with ethanol (100 mM) or media (control), and ROS generation was measured at different time points. N≥3 for all measures, *p<0.05 for comparison with untreated cells.

We created a stable line of MLE 12 cells expressing an oxidant sensitive GFP probe localized to the mitochondrial matrix. We treated these cells with increasing doses of ethanol (0-100 mM) or TBHP (positive control) for 4 hours or high dose ethanol (100 mM) for different durations and measured ROS generation using flow cytometry (Figure 3A). Alcohol did not cause any appreciable ROS generation in lung epithelial cells even when administered at the highest concentration (100 mM) for 4 hours (Figure 3B).

### Alcohol increases levels of adenosine in mouse lungs and in the culture media from alveolar epithelial cells

**Figure 4:**
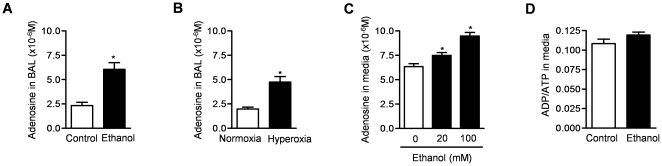
Alcohol increases adenosine levels in the lung and induces the release of adenosine from primary alveolar epithelial cells. (**A**) We treated mice with either ethanol (20% v/v, 4 g/kg, i.p.) or control vehicle (sterile water) and adenosine levels in BAL fluid were measured at 4 hours using HPLC. (**B**) We exposed mice to either hyperoxia (>95% O_2_) or normoxia (21%) for 72 hours and then measured adenosine levels in BAL fluid. Primary rat alveolar epithelial cells were treated with ethanol and (**C**) adenosine levels in the culture media (**D**) ADP and ATP levels in cell lysates were measured after 4 hours. N ≥ 3 for all measures, *p<0.05 for comparison with untreated cells.

We treated C57BL/6 mice with ethanol (20% v:v, 4 g/kg, i.p.) or control vehicle (sterile water) and 4 hours later collected BAL fluid in the presence of inhibitors of adenosine metabolism (erythro-9-(2-hydroxy-3-nonyl) adenine [EHNA], 2.5 µM and dipyridamole 250 µM) for measurement of adenosine using HPLC [32], [33]. We observed significantly higher levels of adenosine in the BAL fluid from the ethanol compared to control mice (Figure 4A). We also measured adenosine levels in the BAL fluid from mice exposed to hyperoxia for 72 hours and found similar increases in adenosine levels (Figure 4B). To determine whether alcohol can lead to an increase in extracellular adenosine levels in the lung, we treated primary rat alveolar type II cells with ethanol (0–100 mM) and 4 hours later measured adenosine levels in the culture media and ADP and ATP levels in the cell lysates. Ethanol treatment caused a dose-dependent increase in adenosine levels in the culture media (Figure 4C). Ethanol did not significantly alter ADP/ATP levels in primary rat AEC (Figure 4D).

### Alcohol-induced reduction in Na,K-ATPase is mediated via adenosine A1 receptor

**Figure 5:**
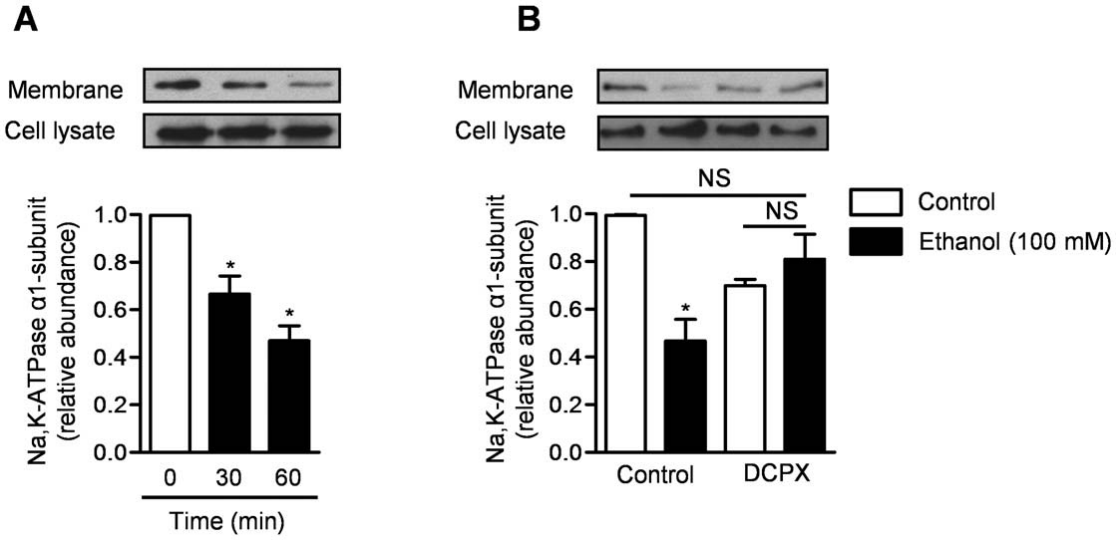
Alcohol-induced reduction in Na,K-ATPase is mediated via ADORA1. Membrane abundance of the α_1_-subunit of Na,K-ATPase was evaluated via immunoblotting membrane proteins (biotin pulldown) in primary rat AEC. (**A**) Cells were treated with an ADORA1 agonist (CPA, 10^−5^M) at different time points. (**B**) Cells were treated with ethanol (100 mM) or control vehicle (sterile water) in the absence or presence of an ADORA1 antagonist (DCPX, 10^−5^M). N≥3 for all measures, *p<0.05 for comparison with untreated cells.

We treated primary rat AEC with an ADORA1 agonist (2-chloro-N^6^-cyclopentyladenosine (CPA) 10^−5^M) and immunoblotted the basolateral membrane fraction for Na,K-ATPase α1-subunit over time. Stimulation of ADORA1 caused a progressive reduction in the membrane abundance of Na,K-ATPase (Figure 5A) similar to what we observed with ethanol treatment. To determine whether alcohol induced reduction in Na,K-ATPase is mediated via ADORA1, we treated primary rat AEC with ethanol (100 mM) or control in the absence or presence of an ADORA1 antagonist (DCPX, 10 ^−5^ M). Administration of DCPX inhibited the alcohol-induced reduction in the membrane abundance of Na,K-ATPase (Figure 5B) suggesting the alcohol-induced reduction in active Na+ transport is mediated via ADORA1.

### Alcohol-induced reduction in Na,K-ATPase is mediated via phosphorylation of AMPK

**Figure 6:**
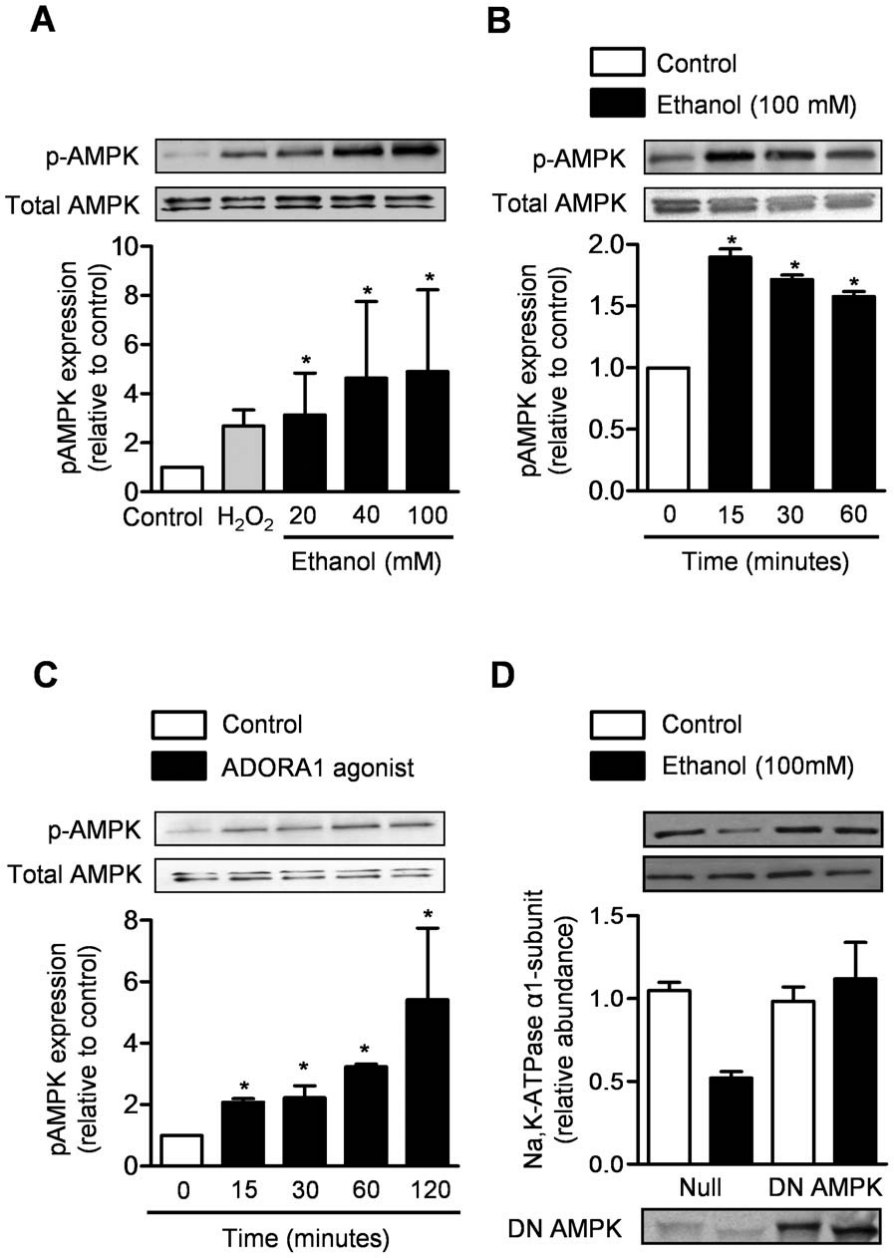
Alcohol-induced reduction in Na,K-ATPase is mediated via phosphorylation of AMP-activated protein kinase. Immunoblotting for phosphorylated AMPK (p-AMPK) and total AMPK was performed in cell lysates from (**A**) AEC 15 minutes after treatment with different concentrations of ethanol, H_2_O_2_ (positive control) or media (negative control), (**B**) at different time points after the administration of ethanol (100 mM) or media or (**C**) after treatment with a ADORA1 agonist (CPA, 10^−5^M) or control (DMSO). (**D**) Membrane abundance of the α_1_-subunit of Na,K-ATPase was evaluated via immunoblotting in rat AEC transfected with an adenovirus that expresses no transgene (Adnull) or that expresses a dominant-negative, kinase-dead variant of the AMPK α_1_ subunit (AdDN AMPK) to generate a non-functional AMPK. N≥3 for all measures, *p<0.05 for comparison with untreated cells.

We treated primary rat AEC with different doses of ethanol (0–100 mM) and immunoblotted the cell lysates for phopho-AMPK (pAMPK) and total AMPK at multiple time points in the first hour after exposure. Ethanol treatment caused phosphorylation of AMPK at all doses (Figure 6A). The phosphorylation occurred as early as 15 minutes after treatment with ethanol (100 mM) (Figure 6B). To determine the effects of adenosine and ADORA1 on AMPK, we treated AEC with the ADORA1 agonist (CPA, 10^−5^M) and immunoblotted the cell lysates for pAMPK and total AMPK at multiple time points in the first hour after exposure. Similar to ethanol treatment, the administration of an ADORA1 agonist (CPA, 10^−5^M) caused phosphorylation of AMPK (Figure 6C).

To determine whether alcohol-induced phosphorylation of AMPK is required for the alcohol-induced reduction in Na,K-ATPase, we transfected primary rat AEC with an adenovirus that expresses no transgene (AdNull, 20 pfu/cell) or that expresses a hemagglutinin (HA)-tagged adenovirus expressing a dominant-negative, kinase-dead (K45R) variant of the AMPK α_1_ subunit (Ad-HA-DN AMPK-α [AdDN AMPK]; 20 pfu/cell) to generate a non-functional AMPK.[26], [34], [35] We did not observe an alcohol-induced reduction in Na,K-ATPase abundance in cells infected with the DN AMPK (Figure 6D). As increase in AMP is an important stimulus for AMPK activation, we measured intracellular levels of adenine nucleotides (AMP, ADP, ATP), which were not different in alcohol treated cells compared to control (Figure 4C).

## Discussion

We found that alcohol worsens survival of mice exposed to hyperoxia, a well-recognized murine model of acute lung injury. Alcohol administration was accompanied by an increase in lung levels of adenosine and by a reduction in the rate of alveolar fluid clearance. We found that alcohol treatment caused cultured primary rat alveolar type II cells to release adenosine into the media. In these cells, the administration of alcohol or adenosine was sufficient to induce a reduction in the plasma membrane abundance of Na,K-ATPase α1-subunit, suggesting endocytosis of the pump. The alcohol-induced endocytosis of the Na,K-ATPase was inhibited when the cells were treated with an ADORA1 antagonist. The administration of alcohol induced the phosphorylation of AMPK which was required for endocytosis of the Na,K-ATPase induced by both alcohol and pharmacologic activation of ADORA1. These findings are consistent with the inhibitory effect of activation of AMPK on the abundance and activity of the Na,K-ATPase reported by our group and others [26], [34], [35].

Adenosine is generated in all living cells as a byproduct of the metabolism of ATP, a major source of biochemical energy for the cell. Large, channel-forming extracellular nucleoside transporters regulate the cellular reuptake of adenosine. In the brain, alcohol increases the levels of extracellular adenosine by inhibiting its reuptake through this transporter [17], [19], [20], [36]. Extracellular adenosine engages specific receptors on the same or adjacent cells in an autocrine or paracrine fashion with important consequences for cellular function. Adenosine signals through four distinct G protein-coupled receptors, type 1 (ADORA1), 2a (ADORA2a), 2b (ADORA2b), and 3 (ADORA3). In most cell systems, engagement of adenosine with ADORA2a and ADORA2b activate adenylyl cyclase via activation of stimulatory G proteins Gs, which increases cAMP; whereas ADORA1 and ADORA3 inhibit adenylyl cyclase via activation of inhibitory G proteins Gi, which decrease cAMP levels [37]. Each cell may have more than one type of these receptors. We have previously shown that while all four adenosine receptors are expressed on the apical surface of alveolar epithelial cells from rats and mice, ADORA1 is the most highly expressed receptor in these cells [21].

There is growing evidence for adenosine as a key player in the pathogenesis of acute lung injury [38]. Adenosine levels are increased during different models of acute lung injury [39], [40]. We found that similar to other models of acute lung injury, hyperoxia also increases lung levels of adenosine. However, due to the number of types of adenosine receptors and their ubiquitous distribution, the role of adenosine and contribution of specific adenosine receptors to the development of lung injury are not completely understood. Studies suggested that activation of ADORA2a and ADORA2b may attenuate acute lung injury and therefore these receptor types may serve as potential therapeutic targets for the treatment of lung injury [37], [41]. In contrast to beneficial effects of adenosine via activation of ADORA2a and ADORA2b activation, a more recent study showed that inhibition of adenosine via chronic or acute high dose administration of caffeine, which is a non-selective antagonist of adenosine receptors attenuates lung injury and inflammation in mice [42]. Furthermore, other studies evaluating the role of ADORA1suggested that inhibition of the effects of adenosine via ADORA1 may attenuate lung injury [39], [43].

We found that systemic administration of alcohol to mice increased lung levels of adenosine, reduced alveolar fluid clearance and worsened survival during exposure to hyperoxia. We have previously reported that the administration of adenosine reduced alveolar fluid clearance via a mechanism that required ADORA1 [21]. We and others have shown that strategies that improve alveolar fluid clearance are associated with improved survival in mice and rats exposed to hyperoxia and reduced levels of alveolar fluid clearance are associated with poor clinical outcomes in patients with acute lung injury [9]–[16]. Therefore, we speculate that the activation of ADORA1 in response to alcohol may be responsible for the observed reduction in alveolar fluid clearance and the worsened survival in mice exposed to hyperoxia. However, our results do not directly make this link. Because activation of ADORA1 and other adenosine receptors might have differential effects on inflammatory and epithelial cells in the lung, tissue specific loss of function studies in mice will be required to address this question. Nevertheless, our results suggest a potentially important mechanism by which alcohol might worsen lung injury. As alcohol induces an increase in adenosine levels in the liver and brain similar to the increase we observed in the lung, our results suggest a common pathway by which alcohol activates signaling pathways to impair the function of multiple organs and suggest a common target for therapy.

The AMP-activated protein kinase (AMPK) is a highly conserved Ser/Thr kinase, which is activated by phosphorylation in response to metabolic conditions where substrates for metabolism are limited, for example fasting or exercise. Substrates of AMPK include proteins required to mobilize stored substrates (glycogen and fats) and downregulate biosynthetic processes (lipogenesis, protein synthesis), thereby coordinating an adaptive response to metabolic stress [44]. In the mammalian epithelium, AMPK has been reported to inhibit the epithelial Na^+^ channel (ENaC) and the cystic fibrosis transmembrane conductance regulator chloride channel [45], [46]. We have previously found that the activation of PKCζ by AMPK is required for endocytosis of the Na,K-ATPase in response to hypoxia and hypercapnia [26], [27], [35], [47]. Here we found that exposure of lung epithelial cells to alcohol resulted in AMPK activation and overexpression of a dominant negative AMPK prevented the alcohol-induced endocytosis of the Na,K-ATPase. AMPK is a heterotrimeric protein composed of α, β and γ subunits [44], [48]. All three isoforms of the γ subunit contain domains that bind AMP and ATP. Binding of AMP activates the kinase by promoting its phosphorylation at Thr-172 by its upstream kinase LKB1, protecting it from phosphatases and by direct allosteric activation [44], [48]. High levels of ATP compete with AMP for the AMP binding sites in the γ subunits [49]. We were unable to detect an increase in AMP levels in response to alcohol suggesting that alternate pathways are responsible for the alcohol and adenosine mediated activation of AMPK. For example, calmodulin-dependent protein kinase kinase (CaMKK) can phosphorylate AMPK in response to increases in cytosolic calcium [50] and we have reported that these calcium transients are required for the AMPK-dependent phosphorylation and endocytosis of the Na,K-ATPase in response to increases in extracellular CO_2_ concentrations [26].

Other groups have observed a reduction in the levels of reduced glutathione in the lungs of alcohol exposed rodents and in patients with alcoholism who develop ARDS [51]–[53]. As we have previously observed that mitochondrially generated ROS can induce PKCζ-mediated phosphorylation of the Na,K-ATPase resulting in its endocytosis, we hypothesized that alcohol might induce endocytosis of the Na,K-ATPase through a similar mechanism [27], [35]. However, we were unable to detect an increase in ROS in response to alcohol. It may be that the reactive oxygen species responsible for the reduced glutathione levels observed in the lungs of humans and animals exposed to alcohol are derived from inflammatory or other cells within the lung or that metabolic changes induced by longer durations of ethanol exposure prevent the regeneration of glutathione.

One-half of the U.S. population consumes alcohol on a regular basis and it is estimated that about 15–20 million people are alcoholics [54], [55]. The Centers for Disease Control and Prevention reported approximately 76,000 deaths and more than 2.3 million years of potential life lost attributable to alcohol abuse in the United States alone in 2001 [56]. Chronic alcohol abuse independently increases the incidence of ARDS by two- to four-fold and is associated with increased mortality related to multisystem organ failure in patients with ARDS [4], [5], [54], [55], [57], [58]. As patients with ARDS whose alveolar fluid clearance is impaired are more likely to die, our results suggest that an alcohol-induced increase in lung adenosine, which acts through ADORA1 and AMPK to cause endocytosis of the Na,K-ATPase, might impair alveolar fluid clearance and contribute to poor outcomes. Our results further suggest therapies that target ADORA1 and/or AMPK may potentially be beneficial in the treatment of alcohol abuse related ARDS.
